# Subcircuit-specific neuromodulation in the prefrontal cortex

**DOI:** 10.3389/fncir.2014.00054

**Published:** 2014-06-05

**Authors:** Nikolai Dembrow, Daniel Johnston

**Affiliations:** Center for Learning and Memory, The University of Texas at AustinAustin, TX, USA

**Keywords:** neuromodulation, projection neurons, prefrontal cortex

## Abstract

During goal-directed behavior, the prefrontal cortex (PFC) exerts top-down control over numerous cortical and subcortical regions. PFC dysfunction has been linked to many disorders that involve deficits in cognitive performance, attention, motivation, and/or impulse control. A common theme among these disorders is that neuromodulation of the PFC is disrupted. Anatomically, the PFC is reciprocally connected with virtually all neuromodulatory centers. Recent studies of PFC neurons, both in vivo and ex vivo, have found that subpopulations of prefrontal projection neurons can be segregated into distinct subcircuits based on their long-range projection targets. These subpopulations differ in their connectivity, intrinsic properties, and responses to neuromodulators. In this review we outline the evidence for subcircuit-specific neuromodulation in the PFC, and describe some of the functional consequences of selective neuromodulation on behavioral states during goal-directed behavior.

## Introduction

The prefrontal cortex (PFC) guides experience-driven, goal-directed behavior. Hallmarks of PFC damage include incapacity to suppress impulsive responses and inability to switch strategies when a previously learned rule is no longer successful (Milner, 1963; Shallice and Burgess, 1991; Aron et al., 2004). Similar deficits are observed in non-human primates performing rule-guided tasks after the PFC is lesioned or inactivated (Brozoski et al., 1979; Dias et al., 1996). Although rodents do not exhibit goal directed behaviors as sophisticated as those observed in primates, disrupting functionally analogous regions of the rodent PFC impairs performance in a variety of tasks designed to test executive function: temporal control (Risterucci et al., 2003; Narayanan and Laubach, 2006; Narayanan et al., 2013), attention (Broersen and Uylings, 1999; Chudasama et al., 2005; Kahn et al., 2012), working memory (Floresco et al., 1997; Dias and Aggleton, 2000; Lee and Kesner, 2003), and strategy shifting (Ragozzino et al., 1999a,b, 2003; Rich and Shapiro, 2007, 2009). Different components of PFC function may be mediated by different PFC subregions (well reviewed in Robbins, 1996; Uylings et al., 2003; Kesner and Churchwell, 2011). Elucidating the precise cellular constituents and mechanism(s) underlying PFC function, and how it exerts top-down control over other brain regions, remains an important area of exploration.

One critical component for PFC function is the contribution of neuromodulatory inputs. How neuromodulation contributes to the executive control of goal directed behavior has been largely examined on two separate levels: actions of neuromodulators on generic neurons and/or synapses within the PFC, and the effects of neuromodulators on network activity in conjunction with behavioral performance. The goal of this review is to begin to bridge these two levels of analysis by detailing recent advances in mapping out connectivity, neuromodulatory responses and the intrinsic properties of different classes of projection neurons in the rodent PFC.

## Neuromodulation and the prefrontal cortex

**Figure 1:**
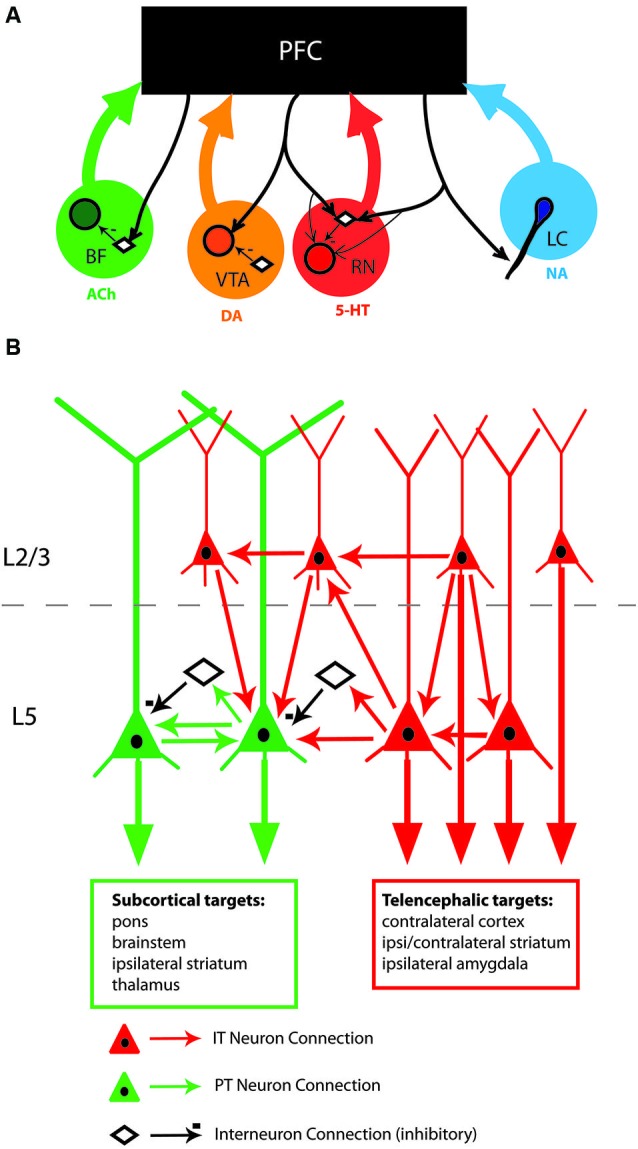
**(A)** Schematic of descending connections from PFC to neuromodulatory centers showing the cellular targets of PFC fibers. Noradrenaline (NA) is released from terminals projecting from the locus coeruleus (LC). Cholinergic (ACh) terminals originate from the basal forebrain. Serotonin (5-HT) terminals originate from the medial and dorsal raphe nuclei (RN). Depending on the species of animal, terminals from the ventral tegmental area (VTA) and/or the substantia nigra (SN) are sources of dopamine (DA) within the PFC (for a review, see Berger et al., 1991). PFC fibers connect onto neuromodulator-synthesizing projection neurons (shaded circles), inhibitory interneurons (open diamonds), or both. In the case of locus coeruleus, PFC inputs synapse onto the dendrites of noradrenergic neurons. **(B)** Schematic of identified connections within the rodent mPFC. Pyramidal tract (PT, green) and intratelencephalic (IT, red) neurons are embedded within the PFC network differently. PT neurons are confined to L5/6 while IT neurons are found throughout L2-6. PT neurons receive inputs from PT, IT, and inhibitory interneurons. IT neurons receive only inputs from other IT neurons. L5 PT and IT neurons are shown in proximity for the purposes of the schematic, in the tissue they are interspersed amongst one another. Abbreviations: BF, basal forebrain; ACh, acetylcholine; VTA, ventral tegmental area; DA, dopamine; RN, raphe nuclei; 5-HT, serotonin; LC, locus coeruleus; NA, noradrenaline.

The efficacy by which the PFC drives behavior is highly sensitive to the actions of neuromodulators. Best studied among these include noradrenaline (NA), acetylcholine (ACh), serotonin (5-HT), and dopamine (DA). Other neuromodulators (histamine, adenosine, and many neuropeptides) can also alter PFC function, but for the purposes of this mini review we will focus on these four. The primary source of neuromodulators in the PFC is from terminals originating from subcortical neuromodulatory systems (Figure 1A). Infusing neuromodulators or their receptor agonists/antagonists directly into the PFC changes behavioral performance (Febvret et al., 1991; Broersen et al., 1995; Ragozzino and Kesner, 1998; Mao et al., 1999; Wall et al., 2001; Winstanley et al., 2003; Bang and Commons, 2012; Yang et al., 2013). Optimal PFC function occurs within a tight range of neuromodulatory action: both too little and too much of a given neuromodulator will impair task performance (Broersen et al., 1995; Zahrt et al., 1997; Ragozzino and Kesner, 1998; Mao et al., 1999; Granon et al., 2000; Wall et al., 2001; Winstanley et al., 2003; Vijayraghavan et al., 2007; Wang et al., 2007; Yang et al., 2013).

Anatomically, the PFC is reciprocally connected with these neuromodulatory centers (Figure 1A). While none of the neuromodulatory centers exclusively targets the PFC, there is a topographical organization to these outputs (Berger et al., 1991; Bang et al., 2012; Zaborszky et al., 2013). For example individual LC inputs, but not BF inputs, preferentially target either the ventral mPFC or dorsal mPFC (Chandler and Waterhouse, 2012). The PFC neuromodulatory inputs may be specialized in some cases. The PFC is one of few cortical regions that receive input from both the medial and dorsal portions of the RN (Bang et al., 2012). Similarly, dopaminergic fibers originate from the VTA and SN in the rodent PFC (Berger et al., 1991). The density of cholinergic fibers, and of the enzyme acetylcholinesterase (the enzyme responsible for removing extracellular acetylcholine), is densest in the mPFC, suggesting that cholinergic input is particularly tightly controlled there (Werd et al., 2010; Zaborszky et al., 2012). It is important to note that in addition to the neuromodulatory substance each center produces, some of their projections also can contain fast inhibitory (GABAergic) and/or excitatory (glutamatergic) transmitters (Febvret et al., 1991; Hur and Zaborszky, 2005; Bang and Commons, 2012; Chandler and Waterhouse, 2012). Thus, the effect of neuromodulatory centers on the PFC may act on multiple time scales.

In addition to receiving input from subcortical neuromodulatory systems, glutamatergic outputs from PFC selectively target specific neuron populations within each neuromodulatory center. In the VTA, prefrontal inputs synapse upon the dopaminergic neurons that project back to the PFC, but not with neurons projecting to the accumbens. Conversely, prefrontal inputs synapse onto GABAergic neurons projecting to nucleus accumbens, but not those projecting to the PFC (Carr and Sesack, 2000). Prefrontal inputs to LC synapse onto the dendrites of noradrenergic neurons in the peri-LC region (Luppi et al., 1995). In the dorsal RN, prefrontal inputs synapse primarily onto GABAergic interneurons, although they also synapse on serotonergic neurons as well (Jankowski and Sesack, 2004; Commons et al., 2005). Similarly prefrontal projections to the BF synapse onto inhibitory parvalbumin-positive interneurons, but not cholinergic projection neurons, in the horizontal limb of the BF (Zaborszky et al., 1997). Consistent with selective innervation of neuromodulatory centers, PFC stimulation promotes burst firing in VTA and increased activity in LC, but inhibits firing in dorsal raphe nuclei (DRN) and BF (Overton et al., 1996; Tong et al., 1996; Jodo and Aston-Jones, 1997; Jodo et al., 1998; Celada et al., 2001). As such, the PFC is able to regulate its own neuromodulatory input by driving or inhibiting subcortical centers.

In addition to regulating its own neuromodulatory input, the PFC may also alter the output of neuromodulatory centers to other brain areas. This provides an interesting means by which the PFC might exert a more global “top-down” control of behavior. A small population of PFC neurons may be responsible for this output, as individual neurons within the PFC innervate more than one neuromodulatory center. For instance, a small population of PFC neurons project to both the RN and the VTA (Gabbott et al., 2005; Vázquez-Borsetti et al., 2009, 2011). Similarly, a subset of PFC neurons project to both RN and LC (Lee et al., 2005). The extent to which these projections represent a means to exert top-down control over other brain regions represents an exciting area of exploration for future studies.

## PFC projection neurons

By using optogenetic stimulation in vivo, several studies have demonstrated that the PFC can alter behavior. In one important study, Warden et. al. tested the effects of optogenetically driving the PFC during a forced-swim test (Warden et al., 2012). Driving PFC output to the DRN promoted active escape, while driving PFC output to the lateral habenula inhibited escape behavior. These results suggest that different subsets of PFC output neurons drive distinct, even mutually antagonistic, behaviors. Other groups have shown that PFC output to the amygdala, striatum, and DRN shift behavioral output (Challis et al., 2014; Vialou et al., 2014). But what is the identity of these output neurons, and what electrophysiological properties and connectivity patterns do they exhibit?

To better understand how the PFC *exerts top-down control* over downstream targets, it is useful to identify and characterize the neurons that provide output from the PFC. Most of this work has been done in the rodent medial prefrontal cortex. Cytoarchitectonically, the rodent PFC differs from the primate PFC in that it is agranular cortex, meaning that it lacks a granule-cell layer 4. Despite this, supragranular pyramidal neurons (in layers 2–3) can be demarcated from infragranular pyramidal neurons (in layers 5–6) by a band of thalamocortical fibers in deep layer 3 (Kubota et al., 2007; Cruikshank et al., 2012; Hirai et al., 2012).

Output neurons of the PFC are broadly divided into two categories: (1) pyramidal tract, or PT neurons, and (2) intratelencephalic, or IT neurons (Molnár and Cheung, 2006; Shepherd, 2013). PT neurons project subcortically via the pyramidal tracts projecting to ipsilateral striatum, thalamus, and/or brainstem. PT neurons are located within the infragranular layers. Unlike motor and sensory cortex, both PT and IT L5 neurons in the PFC are distributed throughout L5A and L5B (Dembrow et al., 2010; Hirai et al., 2012; Ueta et al., 2013, but see Cowan and Wilson, 1994). IT neurons are present in both supragranular and infragranular layers of PFC. They make long-range projections to ipsilateral perirhinal cortex, amygdala and striatum, as well as to the contralateral striatum and cortex (Gabbott et al., 2005; Hirai et al., 2012). The IT and PT categories express disparate transcription factors during development that guide their different long-range projections (Molyneaux et al., 2007, 2009; Fame et al., 2011). Recently, it has become evident that L5 PT and IT neurons within rodent PFC possess distinct intrinsic properties, local connectivity, and long-range inputs. Although most of these differences have been characterized in rodents, different categories of PFC pyramidal neurons are also present in humans and non-human primates (Foehring et al., 1991; Tasker et al., 1996; Chang and Luebke, 2007). PT and IT neuron categories can be further subdivided into groups based on gene expression, specific projection targets and laminar distribution. IT neurons are particularly diverse (Molyneaux et al., 2009). PT neurons project to the thalamus or spinal cord depending upon whether they are in L5A or 5B, respectively (Hirai et al., 2012; Ueta et al., 2013).

PT and IT neurons are connected within the PFC differently (Schematic Figure 1B). Most of this work has been done by Kawaguchi and colleagues in the cortical subregion immediately dorsal to, or within, the most dorsal part of mPFC. L2/3 IT and L5 IT neurons receive inputs from other IT neurons, but very infrequently from PT neurons (Morishima and Kawaguchi, 2006). In contrast, PT neurons receive inputs from both L2/3 and L5 IT neurons, as well as from other PT neurons. PT neurons exhibit higher rates of reciprocal connections (where two PT neurons mutually excite one another) than do IT neurons (Morishima and Kawaguchi, 2006; Morishima et al., 2011). Paired recordings of PT-like and IT- like neurons (categorized by their morphology) suggest that PT to PT connections display more synaptic augmentation (Wang et al., 2006). Such synaptic specializations may underlie the robustness of behavior-dependent persistent activity of neurons in the PFC, as compared with other cortical areas (Hempel et al., 2000; Wang et al., 2006, 2008). PT and IT neurons receive different inhibitory inputs from local interneurons as well. PT and IT neurons seem to be equivalently connected to fast spiking interneurons (Otsuka and Kawaguchi, 2013), however PT neurons receive stronger inhibition from parvalbumin-positive fast spiking interneurons (Lee et al., 2014). Therefore, PT neurons may represent a final convergence point for numerous local excitatory and inhibitory synaptic inputs.

**Figure 2:**
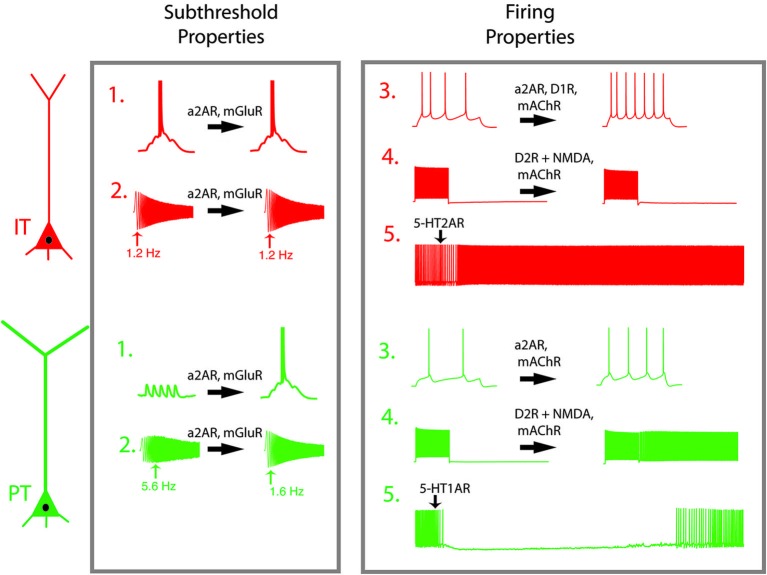
**Neuromodulators shift the dynamic properties of L5 PFC projection neurons**. Pyramidal tract (PT, green) and intratelencephalic (IT, red) have different response profiles in their subthreshold (1–2) and spiking (3–5) properties. (1) Summation of synaptic inputs. In IT neurons, excitatory potentials spread out in time summate to trigger action potentials, while in PT neurons temporal summation is limited by intrinsic membrane properties. (2) While IT neurons respond preferentially to low frequency signals (1–2 Hz), PT neurons respond preferentially to theta frequency oscillatory input. (3) In response to a step current injection, PT neurons produce fewer action potentials than do IT neurons. (4) When synaptic activity is blocked, both neurons respond to depolarizing current steps, but return to quiescence once the stimulus is removed. In the presence of D2R activation or mAChR activation, PT neurons remain persistently active after the stimulus is removed. (5) A brief application of serotonin has opposing effects on active PT and IT neurons. Abbreviations: mAChR, muscarinic acetylcholine receptor; a2AR, alpha-2A-adrenergic receptor; D1R, dopamine subtype 1 receptor; D2R, dopamine subtype 2 receptor; mGluR, metabotropic glutamate receptor; 5-HT1A, serotonin subtype 1A receptor, 5-HT2A, serotonin subtype 2A receptor; NMDAR, NMDA receptor. Effects of neuromodulators are adapted from Dembrow et al. (2010); Avesar and Gulledge (2012); Gee et al. (2012) and Seong and Carter (2012).

Equally important to the connections they make and receive, PT and IT neurons exhibit subpopulation-specific intrinsic electrophysiological properties. Such differences cause PT and IT neurons to respond to time-varying signals differently (Dembrow et al., 2010). When injected with a sinusoidal current with increasing frequency, PT neurons respond most strongly in the theta-frequency range (4–10 Hz), while IT neurons respond optimally to slower (<2 Hz) signals (Figure 2). The distinct subthreshold physiological properties of PT and IT neurons are consistent with differences in the hyperpolarization-activated cyclic nucleotide gated cation current (*h*-current) in these neurons. Blocking *h*-current changes the subthreshold properties of both neuron types, abolishing differences in the time-dependent membrane filtering both at the soma and dendrite (Dembrow et al., 2010; Kalmbach et al., 2013). In the apical dendrites, where *h*-channels are preferentially targeted in pyramidal neurons in the hippocampus and somatosensory cortex (Magee, 1999; Williams and Stuart, 2000; Berger et al., 2001), subthreshold differences between IT and PT neurons are more pronounced (Kalmbach et al., 2013). As a result of *h*-current related properties, PT neurons integrate dendritic inputs over a narrow time window, and are thus preferentially responsive to coincident inputs. On the other hand, IT neurons summate over wider time windows, allowing them to better integrate nonsynchronous input.

PT and IT neurons in PFC also express different active properties. IT neurons have a lower threshold for action potential initiation, and greater action potential half-width than PT neurons (Dembrow et al., 2010). These differences are also observed in anaesthetized animals in vivo (Cowan and Wilson, 1994). Once driven to spike, PT and IT neurons exhibit differing firing patterns. In response to a long (10 s) square step of current sufficient to drive action potentials depolarization, PT neurons show spike frequency acceleration. In contrast, IT neurons show significant spike frequency accommodation (Morishima and Kawaguchi, 2006; Otsuka and Kawaguchi, 2008; Dembrow et al., 2010). In other cortical regions, the acceleration in spiking in is caused by a “D”-type potassium current (Miller et al., 2008). The source of IT spike accommodation is less clear. Enhancing small conductance calcium-activated potassium channel (SK)-type currents can contribute to spike frequency accommodation (Pedarzani et al., 2005). IT neurons display a pronounced slow afterhyperpolarizations (Kalmbach et al., 2013), which may be partially caused by calcium-sensitive potassium channels (but see Gulledge et al., 2013). Alternatively, differences in accommodation may be caused by *m*-current, sodium-dependent potassium current, sodium pump activity, or differences in the inactivation recovery time of sodium channels that drive the spikes (Schwindt et al., 1989; Santini and Porter, 2010; Gulledge et al., 2013).

The importance of differences in ion channel expression in PT and IT neurons is highlighted by observations that manipulating these ion channels alters working memory performance. Manipulations of *h*-current within the PFC alter working memory task performance in both monkeys and rodents. Removal the hyperpolarization-activated cyclic nucleotide-gated channel 1 (HCN1) subunit from the mPFC impaired performance on a delayed alternation task (Thuault et al., 2013), while *h*-channel blockade, or HCN1 knockdown, improved memory performance (Wang et al., 2007). Similarly, both SK channel and *m*-current blockade can enhance working memory function (Brennan and Arnsten, 2008; Wang et al., 2011). Differences in ion channel expression in prefrontal PT and IT neurons likely contribute to their functional role within executive circuits.

## Projection-specific neuromodulation

PT and IT neurons also respond differently to neuromodulation. Neuromodulators change both subthreshold and suprathreshold responses in PT and IT neurons. In the presence of muscarinic activation, PT neurons display a subtle reduction in their subthreshold resonance (Dembrow et al., 2010). More strikingly, PT neurons shift into a persistent firing-primed state, wherein they respond to a brief suprathreshold input with persistent firing lasting tens of seconds (Figure 2, #4). While cholinergic modulation enhances the afterdepolarization in IT neurons, it causes no change in their subthreshold resonance, results in little, if any, persistent firing. Thus, PT and IT neurons respond to cholinergic input differently. Similarly, metabotropic glutamate receptor group I activation causes both PT and IT neurons to exhibit a slow after depolarization, but causes a long lasting reduction in *h*-related parameters only in PT neurons (Figure 2, #2: Kalmbach et al., 2013). Alpha-2A noradrenergic modulation alters *h*-related properties as well. As a result, noradrenergic and metabotropic glutamate receptor shift PT neurons from preferentially responding to coincident inputs to more broadly tuned integrators, effectively making them similar to IT neurons. Importantly, alpha-2A adrenergic modulation increases the input resistance of both PT and IT neurons, increasing their action potential output in response to depolarization (Figure 2, #3: Dembrow et al., 2010). Similarly, adenosine hyperpolarizes both IT-like neurons PT-like neurons via the A1 receptor, although the amount of hyperpolarization is greater in IT neurons (van Aerde et al., 2013). In all of these cases, the responses of PT and IT neurons to neuromodulatory stimulation are constrained by their differential patterns of ion channel expression.

Alternatively, the difference in neuromodulatory responses is the function of cell-type-specific expression of various receptor subtypes in IT and PT neurons. PT neurons are inhibited by serotonin via 5-HT_1A_ receptors (Figure 2, #5), while IT neurons are excited by serotonin via 5-HT_2A_ receptors (Avesar and Gulledge, 2012). Interestingly, 2A-dependent excitation also occurred in supragranular IT neurons that projected contralaterally, while other L2/3 pyramidal neurons were inhibited by serotonin (Avesar and Gulledge, 2012). Consistent with this, in BAC mice expressing green fluorescent protein driven by 5-HT_2A_ receptor expression in the neocortex was most dense in L5A (Weber and Andrade, 2010), a sublayer enriched with IT-like neurons in sensory and motor cortical regions (Reiner et al., 2003; Anderson et al., 2010; Groh et al., 2010).

Dopaminergic modulation also depends on long-range projection types. Reports on the effects of DA in PFC neurons have been complicated by the diversity of response types, which may be due to several complicating factors: dopamine’s instability, diverse actions on interneurons, effects on glutamatergic transmission, and the diversity of DA receptor subtypes. The recent generation of BAC mice selectively expressing reporter genes in neurons that express different DA receptor subtypes has clarified some of this ambiguity. L5 neurons expressing D1 receptors exhibit the physiological and anatomical hallmarks of IT neurons, while D2 receptor expressing L5 neurons have properties consistent with PT neurons (Gee et al., 2012; Seong and Carter, 2012). Further, D1 agonists enhance the firing responses of IT-like neurons via PKA (Figure 2, #3). Conversely, prolonged optogenetic activation of glutamatergic inputs paired with the D2 agonist quinpirole generates a long-lasting afterdepolarization that can produce persistent firing in PT-like, but not IT-like, projection neurons (Figure 2, #4). It remains less clear whether all IT neurons are D1-receptor positive, or whether they are limited to specific subpopulations of IT neurons (e.g., those projecting to the contralateral cortex versus amygdala). Similarly, all PT neurons may not be D2-receptor positive. An earlier study in rats examining receptor mRNA expression in different projection neurons reported that corticothalamic, corticocortical and corticostriatal neurons express D1 and/or D2 receptors, while D2 receptors are absent from corticopontine, corticospinal, and corticothalamic neurons (Gaspar et al., 1995), a result at odds with data from the BAC mice. Further studies will be needed to clarify these discrepancies, and to test whether the expression of other dopamine receptor subtypes (D3, D4, D5) are segregated by projection subtype.

The importance of selective modulation of IT neurons in PFC has been recently highlighted in several in vivo studies. Mice trained in an operant delay task, where they were trained to nose-poke for food 20 s after a light stimulus, were unable to perform correctly timed responses when D1-positive neurons in the PFC were photoinactivated (Narayanan et al., 2012). Conversely, stimulating the D1-positive neurons enhanced temporal precision of behavior. These data are in line with data that infusion of D1 antagonists into the PFC impairs temporal precision in the same task in rats. There may also be a D1-sensitive, IT-subcircuit important for driving food consumption. Infusion of a D1 antagonist into the PFC alters consumption (Touzani et al., 2010; Nair et al., 2011), while feeding activates D1-positive neurons in the PFC. Optogenetically stimulating them increases food intake, while bilateral inactivating them reduces food intake (Land et al., 2014). The downstream target of these neurons is the ipsilateral amygdala. Combined, these studies suggest that the disparate effects of neuromodulatory transmitters may reflect differential expression of receptor subtype and ionic mechanisms in prefrontal neurons projecting to specific downstream brain regions.

## Future directions

PFC-neuromodulatory circuits are beginning to be mapped at the cellular and subcellular level. Rather than uniformly increasing or decreasing activity, the effect of neuromodulators on prefrontal neurons depends upon their long-range targets. Understanding how these modulatory systems contribute to information flow in the PFC will be important for understanding how the PFC exerts top-down control of behavior. This map, however, represents an initial step towards elucidating how these dynamic and plastic systems function (Marder, 2012). Future studies will need to identify the specific neuron subtypes contributing to mnemonic persistent activity, and how neuromodulatory systems selectively regulate synaptic connections and intrinsic excitability within this network. Most importantly, complex models that take into account differences in connectivity, information processing, and long range connections to downstream targets will be necessary to elucidate how the PFC drives goal-directed behaviors.

## Conflict of interest statement

The authors declare that the research was conducted in the absence of any commercial or financial relationships that could be construed as a potential conflict of interest.
